# A predictive model using left atrial function and B‐type natriuretic peptide level in predicting the recurrence of early persistent atrial fibrillation after radiofrequency ablation

**DOI:** 10.1002/clc.23557

**Published:** 2021-02-08

**Authors:** Zhenni Yang, Min Xu, Chuxu Zhang, Huannian Liu, Xiaoliang Shao, Yuetao Wang, Ling Yang, Junhua Yang

**Affiliations:** ^1^ Department of Cardiovascular Division of The Third Affiliated Hospital of Soochow University Chang Zhou City Jiangsu Province China; ^2^ Department of Cardiovascular Division of The First Affiliated Hospital of Soochow University Su Zhou City Jiangsu Province China; ^3^ Department of Cardiovascular Division of Changzhou Hospital Traditional Chinese Medicine Chang Zhou City Jiangsu Province China; ^4^ Department of Cardiovascular Division of Changzhou Cancer Hospital Affiliated to Soochow University Chang Zhou City Jiangsu Province China; ^5^ Department of Nuclear Medicine of The Third Affiliated Hospital of Soochow University Chang Zhou City Jiangsu Province China

**Keywords:** atrial fibrillation, B‐type natriuretic peptide, circular pulmonary vein ablation, echocardiography, prediction model, recurrence

## Abstract

**Aim:**

A predictive model using left atrial function indexes obtained by real‐time three‐dimensional echocardiography (RT‐3DE) and the blood B‐type natriuretic peptide (BNP) level was constructed, and its value in predicting recurrence in patients with early persistent atrial fibrillation (AF) after radiofrequency ablation was explored.

**Methods:**

A total of 228 patients with early persistent AF who were scheduled to receive the first circular pulmonary vein ablation (CPVA) were enrolled. Clinical data of patients were collected: (1) The blood BNP level was measured before radiofrequency ablation; (2) RT‐3DE was used to obtain the left atrial (LA) time‐volume curve; (3) The clinical characteristics, BNP level and LA function parameters were compared, and logistic regression was used to construct a prediction model with combined parameters; (4) The receiver operating characteristic (ROC) curve was used to examine the diagnostic efficacy of the model.

**Results:**

(1) 215 patients with early persistent AF completed CPVA and the follow‐up. After 3–6 months of follow‐up, the patients were divided into sinus rhythm group (160 cases) and recurrence group (55 cases); (2) The recurrence group showed higher minimum LA volume index, diastolic ejection index, and preoperative BNP (all *p* ≤ .001), while the sinus rhythm group exhibited higher expansion index (PI) and left atrial appendage peak emptying velocity (*p* ≤ .001); (3) In univariate analysis, BNP level had the best diagnostic performance in predicting the recurrence of AF(AUC = 0.703). We constructed a model based on LA function and BNP level to predict the recurrence of persistent AF after CPVA. This combined model was better than BNP alone in predicting the recurrence of persistent AF after CPVA (AUC: 0.814 vs. 0.703, z = 2.224, *p* = .026).

**Conclusion:**

The combined model of LA function and blood BNP level has good predictive value for the recurrence of early persistent AF after CPVA.

## 1 INTRODUCTION

Atrial fibrillation (AF) is one of the most common clinical tachyarrhythmias, and its incidence is relatively high. AF can result in a series of severe public health problems, including increased thromboembolic events, cardiogenic sudden death, and low quality of life.^1^

Early persistent AF is a newly defined term; it refers to the continuous AF that lasts for more than 7 days but less than 3 months.^2^ Circular pulmonary vein isolation (CPVI) is the cornerstone of catheter ablation (CA) therapy for AF.^3^ Many studies have shown that circular pulmonary vein ablation (CPVA) is superior to anti‐arrhythmic drug treatment in terms of maintaining sinus rhythm and improving life quality of AF patients.^4^ European Society of Cardiology (ESC) 2020 recommend that:AF catheter ablation for CPVI should/may be considered as first‐line rhythm control therapy to improve symptoms in selected AF patients.^5^

The role of CPVA in the treatment of persistent AF has been recognized.^6^ The clinical characteristics, such as age, CHA_2_DS_2_‐Vasc score, and the pattern of AF, can help formulate the treatment plan, because they are related to adverse outcomes and recurrence of AF. However, despite these predictive factors, the recurrence rate is still relatively high. Thus, it is necessary to search for candidate markers that can accurately predict the outcome of CPVA.

During the progression of AF, AF patients may have atrial structural remodeling, which is mainly manifested as atrial muscle degeneration, increased fibrosis of atrial muscle and extracellular matrix, etc., which will cause atrial enlargement; moreover, atrial structural remodeling is also the structural basis for AF recurrence after CPVA.^7^ The functions of left atrium mainly include atrial storage, passage, and active contraction. Real‐time three‐dimensional echocardiography (RT‐3DE) is a new method to evaluate left atrial (LA) remodeling. The quantification of atrio‐ventricle by RT‐3DE is highly correlated with the gold standard of cardiac magnetic resonance (CMR) imaging and computed tomography.^8^, ^9^ B‐type natriuretic peptide (BNP) is a structurally related peptide hormone secreted by heart cells. The BNP in AF patients is mainly produced by LA cells. The increase in blood BNP level is related to atrium overload and remodeling. Meta‐analys shows that the increased baseline level of BNP is associated with an increased risk of AF recurrence after CPVA, and it is believed that BNP may be a marker for predicting AF recurrence after CPVA.^10^

It is notable that many of the risk factors that are associated with AF also contribute to AF progression, AF recurrence following ablation, and complications associated with AF. Therefore, the studies that focus on both atrial structural and functional changes can help to evaluate the development, maintenance and progression of AF. In this study, we used RT‐3DE to evaluate the structural remodeling of LA, and constructed a predictive model combining LA function and BNP. We explored the efficacy of the combined model based on ultrasound structural function parameters and blood marker in predicting the recurrence of early persistent AF after CPVA.

## 2 MATERIALS AND METHODS

### 2.1 Object

**FIGURE 1:**
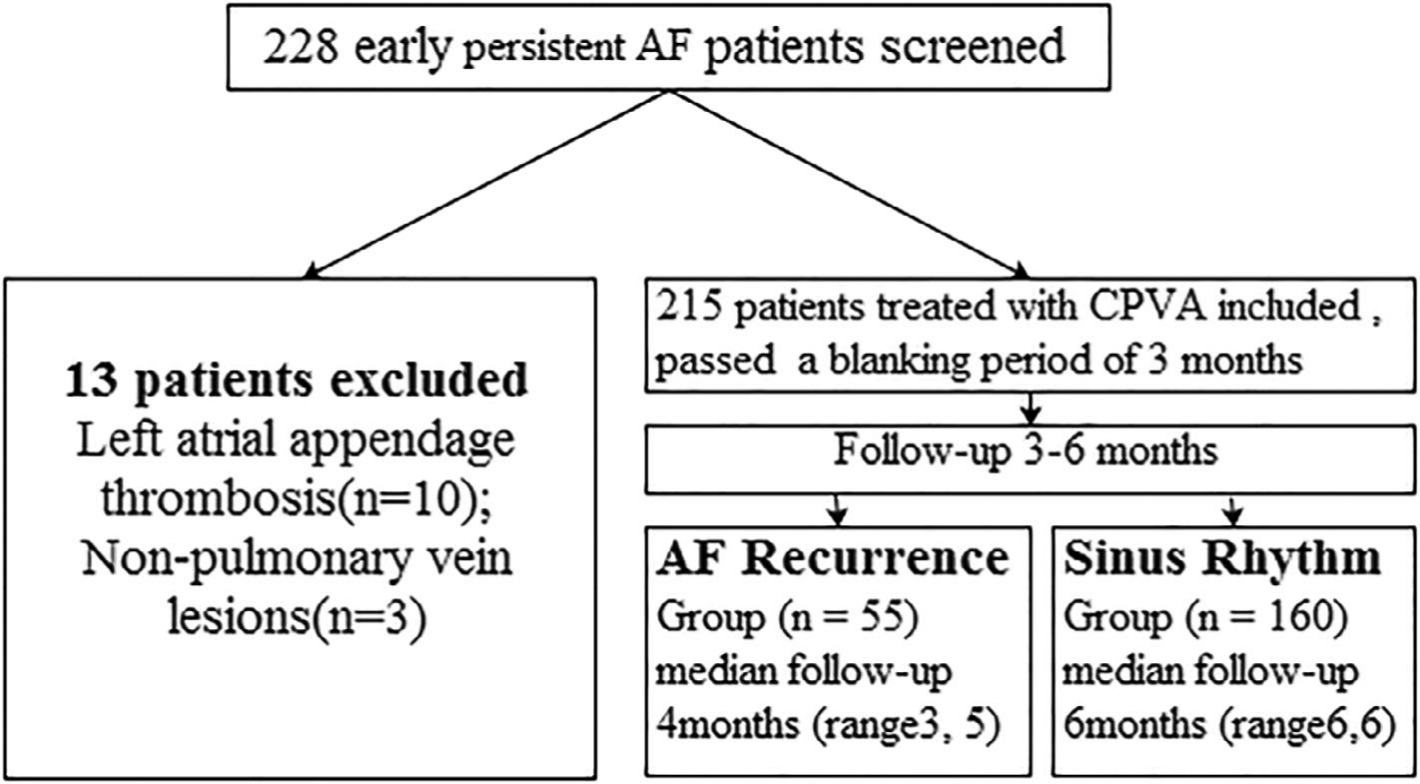
Flowchart of patients selection. AF, atrial fibrillation; CPVA, circular pulmonary vein ablation

A total of 228 patients with early persistent AF who were admitted to The First People's Hospital of Changzhou City, China, from January 2017 to December 2019 and scheduled to receive the first CPVA operation were enrolled in this study, including 188 males and 40 females. Inclusion criteria: (1) Patients with AF recorded by electrocardiogram (ECG) and Holter, and the AF lasted for more than 7 days but less than 3 months; (2) Patients of age 18 to 75 years old; (3) The cardiac function of patients with heart failure returned to cardiac function I (NYHA classification) after treatment; (4) Patients had the ability to understand and sign the informed consent. Exclusion criteria: (1) History of heart disease such as coronary heart disease, valvular disease, and congenital heart disease; (2) Symptoms and signs of cardiac insufficiency did not improve after treatment, and no evidence of cardiac insufficiency in preoperative echocardiography (left ventricular ejection fraction≥50%); (3) Reversible AF, including abnormal thyroid function, acute alcoholism, post‐surgery, and so forth; (4) Patients who had received LA ablation, surgical treatment, atrioventricular node ablation, or other arrhythmias that required ablation; (5) Liver and kidney dysfunction; (6) Respiratory diseases; (7) In the past 3 months, patients had myocardium infarction, percutaneous coronary intervention (PCI), coronary artery bypass graft (CABG), or valve replacement; (8) Patients with hypertrophic obstructive cardiomyopathy; (9) Pregnant or lactating women; (10) Patients with life expectancy <1 year; (11) Recurrence of AF in the first 3 months after ablation. This study complied with the principles of Declaration of Helsinki. All patients signed the written informed consent. The study was approved by the Scientific Ethics Committee. The patient selection process is shown in Figure 1. Informed consents of all patients were obtained from the patients themselves.

### 2.2 Blood sample collection and measurement

According to the standard recommendations from the ESC, on the day before surgery, 2 ml of cubital venous blood was collected from each patient in supine position in the morning while fasting. The blood was put into an anticoagulation test tube with ethylenediamine tetraacetic acid. Within 2 h after collection, the blood sample was centrifuged at 3000 r/min for 10 min, and the supernatant serum was collected for testing. The automatic immunoassay instrument (NniCel Dxl800 Company, USA) was used to measure the serum sample, determined by chemiluminescence method.

### 2.3 Image acquisition and analysis

#### 2.3.1 Echocardiography

The Philips Epiq 7C heart ultrasound system (Netherlands) was used to complete the preoperative transthoracic, transesophageal echocardiography (TEE) and RT‐3DE examinations of all patients. The patient was lying on the left side in a resting state, connected to a 12‐lead ECG for transthoracic image acquisition. The probe was a cardiac probe (X5‐1, X7‐2t) with a depth of 15 cm and an average frame rate of 50 frames/s. Standard M‐shaped, 2D and 3D images were obtained by operating near the sternum and apex. All patients completed TEE and pulse Doppler measurement of left atrial appendage peak emptying velocity (LAAV). Two experienced doctors performed the image acquisition, and five cardiac cycles were continuously acquired. The Philips QLAB software (10.5, Philips Healthcare Royal Philips Electronics, Amsterdam, The Netherlands software pack) was used to analyze the volume and function data. The double‐plane Simpson's method was used to calculate ejection fraction. All TEEs were performed using an X7‐2 probe by a certified echocardiography cardiologist, in order to observe the LA and left atrial appendage (LAA) for thrombosis and spontaneous echocardiographic contrast.

#### 2.3.2 Real‐time three‐dimensional echocardiography

Five cardiac cycles in full‐volume were continuously obtained from apical four‐chamber heart (AP4) for 3D analysis, which required an optimal breath‐hold within 1 day before CPVA. The LA volume‐time curve of the whole cardiac cycle was obtained by RT‐3DE, which yielded the measurements for maximum left atrial volume (LAVmax) and minimum left atrial volume (LAVmin). All left atrial volumes (LAVs) were calibrated to maximum left atrial volume index (LAVImax) and minimum left atrial volume index (LAVImin) according to body surface area. The LA storage function was derived from the following formulas^11^:

$$\text{Expansion index}\;\left(\mathrm{PI}\right)=\left[\left(\text{LAVImax}-\text{LAVImin}\right)/\text{LAVImin}\right]\times 100\%$$

$$\text{Diastolic ejection index}\;\left(\mathrm{DEI}\right)=\left[\left(\text{LAVImax}-\text{LAVImin}\right)/\text{LAVImax}\right]\times 100\%$$

### 2.4 Percutaneous catheter radiofrequency ablation

The combination of CPVA and linear ablation^12^ was performed as follows: the ablation catheter and stimulation catheter were punctured into the right atrium through the femoral vein, and punctured into the left atrium through the atrial septal puncture; then, the CARTO system was used to establish and verify the radio frequency damage, and perform computer tomography to optimize the three‐dimensional reconstruction. The catheter (3.5 mm, Smart Touch Catheter,Biosense Webster Inc., Diamond Bar, CA, USA) with thermocouple needle was used to transmit radio frequency signals. The temperature was 45°C, power was 40 W, and the depth and scope of the damage on local myocardium were about 3–4 mm. To block the two‐way conduction between LA and the pulmonary vein, radiofrequency injury was also applied to the local myocardium around the ipsilateral pulmonary vein, so that the local myocardial voltage was reduced to <0.15 mv. In addition to CPVI, it was necessary to map and ablate the trigger foci outside the pulmonary vein, including tricuspid isthmus line, mitral valve isthmus line, posterior wall top line or top plus bottom (Box) line, anterior wall line, and so forth. The end point of the operation was bidirectional block of the conduction.

### 2.5 Postoperative treatment, follow‐up, and grouping

All patients took new anticoagulants (non‐vitamin K antagonist oral anticoagulants) orally for at least 2 months after the operation, and the electrocardiogram was followed up every day for 3 days after the operation. All patients continued to use an antiarrhythmic drug treatment for 3 months after surgery (amiodarone, 200 mg, Qd; propafenone 150 mg, Tid or morerazine 150 mg, Tid; sotalol 80 mg, Bid). After discharge, patients were followed up for ECG every month, and a 24‐h Holter examination was performed at least once a month. Phone calls and faxes were also used for follow‐up and receiving ECG data. All patients were followed up for at least 3 months and up to 6 months (healing period). AF recurrence that occurred in the first 3 months after the ablation (blanking period) was not included in the present analysis. AF recurrence was defined as a documented episode of any atrial arrhythmias (AF, atrial tachycardia and atrial flutter) lasting ≥30s during the follow‐up period after the3‐month blanking period.^2^ Patient's follow‐up was terminated at the first AF recurrence recorded after the blanking period.

### 2.6 Statistical analysis

R 3.4.3 (http://www.R-project.org/) was used for statistical analysis. Normally distributed data were expressed as $\bar{x}\pm s$, and non‐normally distributed data were expressed as M (P25, P75). The comparisons between groups were performed by independent sample *t* test or Mann–Whitney *U* nonparametric test. Classification data were expressed by frequency or rate (%), and comparison between groups was performed by Pearson chi‐square test or Fisher exact probability method.

Multi‐factor logistic regression was used to establish the prediction model, and the optimal model parameters were selected based on minimum Akaike's information criterion. The Bootstrap resampling (times = 500) method recommended by the TRIPOD report was used to internally verify the model.^13^ For each model, we plotted the ROC curve, and compared the diagnostic efficacy and area under the curve (AUC) of different models using the method from DeLong et al.^14^ *p* < .05 was considered statistically significant.

## 3 RESULTS

Patients without CPVA included 10 patients with left atrial appendage thrombus and 3 patients with atrial fibrillation caused by non‐pulmonary venous disease. A total of 215 patients with early persistent AF completed CPVA treatment. The ablation procedure included wide, encircling pulmonary vein lesions in the 215 patients, roof line in 46 (21.4%), mitral isthmus line in 16 (7.4%), and ablation of complex fractionated electrograms in 15 (7.0%) and cavotricuspid isthmus in 4 patients (1.9%). All patients, including180 males and 35 females, were converted to sinus rhythm during the operation. According to the results of postoperative follow‐up, patients were divided into recurrence group (55 persons, with a recurrence percentage of 25.6%) and sinus rhythm group (160 persons). The comparison of clinical data, blood BNP and echocardiographic parameters between the two groups are shown in Table 1. There recurrence group had higher LAVImin, DEI and BNP, while the sinus rhythm group was higher in PI and LAAV (all *p* < .001).

**TABLE 1:** Comparison of clinical data, BNP and echocardiography parameters

	AF recurrence (*N* = 55)	Sinus rhythm (*N* = 160)	*p* value
Male, *n* (%)	46 (83.6)	134 (73.7)	.984
Age (years)	62.9 ± 9.4	62.8 ± 9.6	.926
BMI > 24(kg/m^2^), *n* (%)	25 (45.5)	74 (46.3)	.919
AF duration, (days)	49.0 (25.0,72.0)	40.5 (22.3,65.8)	.699
Hypertension, *n* (%)	9 (16.4)	43 (26.9)	.116
Diabetes mellitus, *n* (%)	9 (16.4)	36 (22.5)	.335
Hyperlipidemia, *n* (%)	10 (18.2)	24 (15.0)	.577
HR (bpm)	78.0 ± 17.1	77.4 ± 15.8	.822
SCR (umol/l)	62. 0 ± 16.6	60.6 ± 17.7	.610
CHA_2_DS_2_‐Vasc	1.5 ± 1.2	1.7 ± 1.3	.341
LVEF ≥ 52(%), *n* (%)	31 (56.4)	97 (60.6)	.579
LAVImax (ml/m^2^)	41.3 ± 5.3	40.6 ± 5.0	.403
LAVImin(ml/m^2^)	27.7 ± 3.8	25.4 ± 3.8	<.001*
PI (%)	50.0 ± 17.3	61.4 ± 18.8	<.001*
DEI (%)	37.2 ± 7.2	32.5 ± 7.7	<.001*
BNP (pg/mL)	505.3 ± 221.0	352.7 ± 203.4	<.001*
LAAV (cm/s)	34.0 ± 8.3	40.8 ± 11.2	<.001*
Ablation method			
CPVA+Roof‐line ablation, *n* (%)	12 (21.8)	34 (21.2)	.305
CPVA+Mitral isthmus, *n* (%)	5 (9.1)	11 (6.9)	.562
CPVA+CAFEs, *n* (%)	5 (9.1)	10 (6.3)	.540
CPVA+CTI, *n* (%)	1 (1.8)	3 (1.9)	.999

*Note:* * *p* <.05.

Abbreviations: BNP, brain natriuretic peptid; BMI, body mass index; CAFEs, complex fractionated electrograms; CTI, cavotricuspid isthmus; DEI, Diastolic emptying index; LVEF, left ventricular ejection fraction; LAVImax, maximum LA volume index; LAVImin, minimum LA volume index; LAAV, left atrial appendage peak emptying velocity; PI, Expansion index.

**FIGURE 2:**
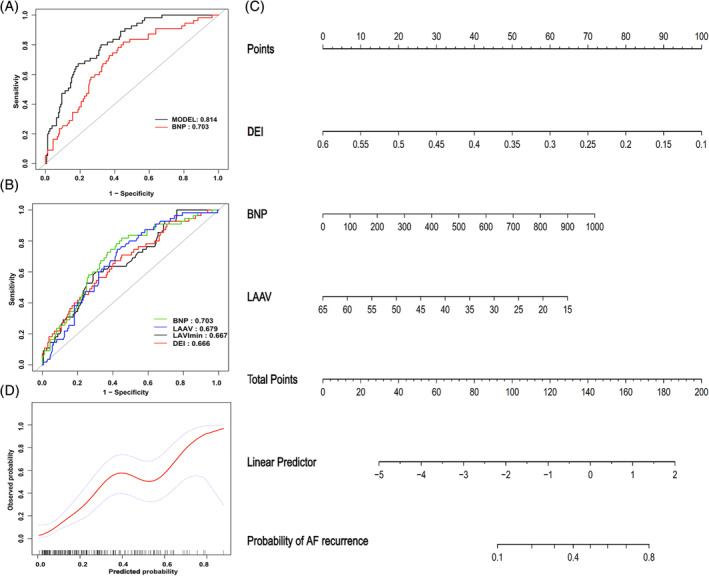
Comparison of the ROC curves of single factors (A) and Model (B) in predicting recurrence of AF;(C) The nomogram of the model in predicting the recurrence of early persistent AF after CPVA; (D) The calibration curve of the combined prediction model in predicting the probability of recurrence in early persistent AF after ablation, The horizontal axis is the predicted incidence, and the vertical axis is the actual incidence of IAC; The black line is the reference line, indicating that the predicted value is the same as the actual value; the red line is the calibration curve, and the blue area is the 95% CI of the predicted value. BNP, B‐type natriuretic peptide; DEI, diastolic ejection index; LAAV, left atrial appendage peak emptying velocity; LAVImin, minimum left atrial volume index

In this study, we first performed single‐factor ROC curve analysis on the factors affecting recurrence after CPVA. The results showed that the four optimal indicators BNP, LAAV, LAVImin, and DEI, among which BNP had the largest AUC (Table 2, Figure 2(A)).

**TABLE 2:** ROC curve to evaluate the efficacy of univariate and predictive model in predicting recurrence of AF

Variable	Cut‐off value	AUC (95%CI)	Specificity	Susceptibility	Accuracy
BNP (pg/mL)	340.950	0.703 (0.625–0.781)	0.550	0.818	0.619
LAAV (cm/s)	38.100	0.679 (0.603–0.755)	0.575	0.746	0.619
LAVImin (ml/m2)	26.833	0.667 (0.587–0.747)	0.713	0.582	0.679
DEI (%)	0.353	0.666 (0.583–0.749)	0.588	0.673	0.609
MODEL	−1.218	0.814 (0.749–0.874)	0.811	0.673	0.776

*Note:* *p < .05.

Abbreviations: BNP, brain natriuretic peptid; DEI, Diastolic emptying index; LAAV, left atrial appendage peak emptying velocity; LAVImin, minimum LA volume index.

Next, we performed multivariate logistic regression analysis, and used all the influential factors in Table 1 as independent variables to construct a predictive model for predicting the recurrence of early persistent AF after CPVA. The equation was: Logit(P) = 2.805–8.953 × DEI + 0.003 × BNP‐0.058 × LAAV.

### 3.1 Comparison of the predictive performance of optimal single factor and combined prediction models

ROC curve analysis was used to compare the diagnostic performance of the optimal single‐factor index BNP and the combined prediction model (Table 2 and Figure 2(B)). The results showed that the prediction performance of the combined prediction model was better than BNP (AUC: 0.814 vs. 0.703), and the differences were significant (z = 2.224, *p* = .026). The nomogram (Figure 2(C)) and the calibration curve (Figure 2(D)) of the combined prediction model indicated a good agreement between predicted value and observed value.

### 3.2 Comparison of the prediction model in different subgroups

**FIGURE 3:**
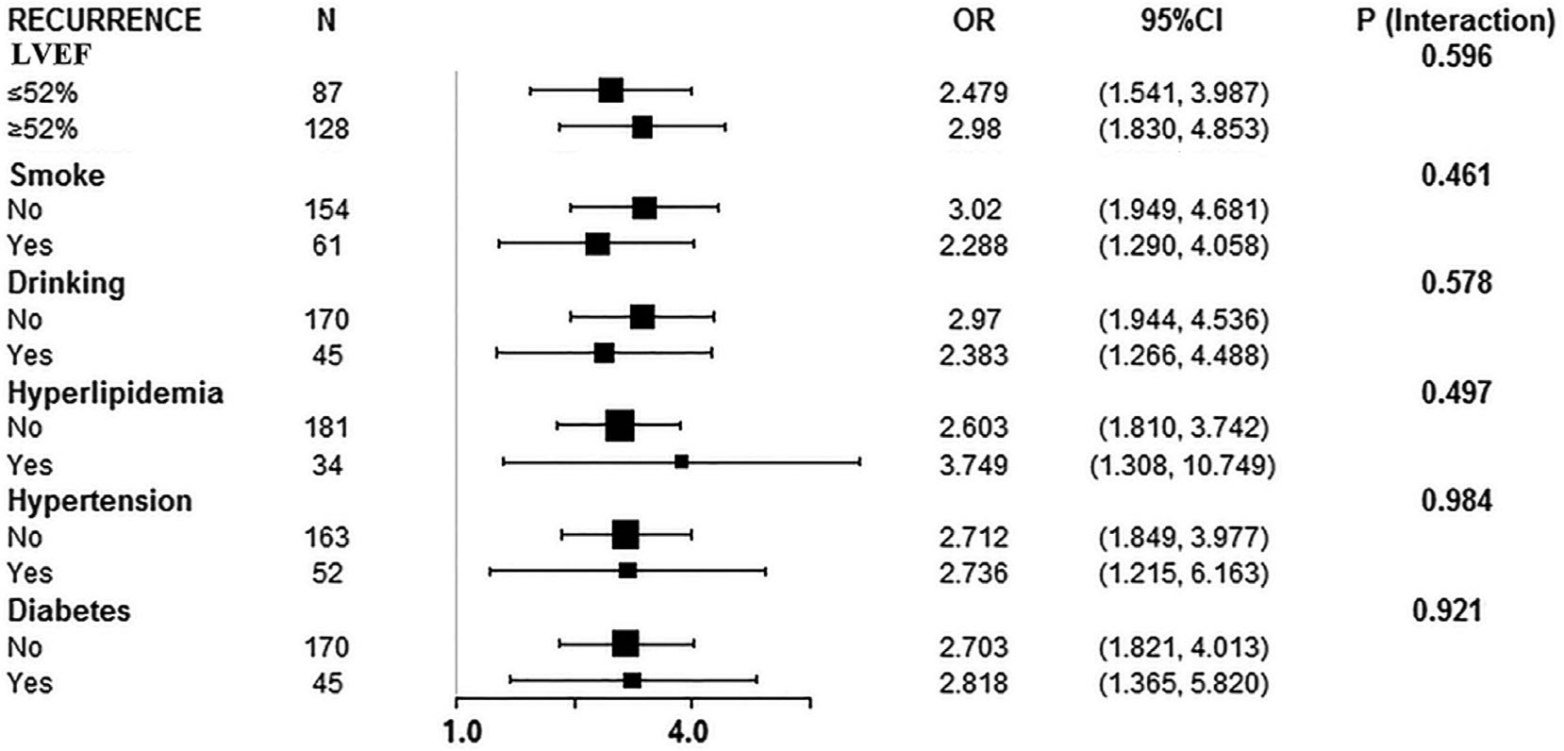
The forest plot of the prediction model in different subgroups. LVEF, left ventricular ejection fraction

After adjusting for age and gender, the multi‐factor predictive model showed consistent trends in subgroups with different left ventricular ejection fraction, smoking, drinking, hyperlipidemia, hypertension, and diabetes history, all of which were risk factors (Figure 3).

## 4 CONCLUSION

In this study, we constructed a combined prediction model based on LA function and blood BNP level for predicting the recurrence of AF in early persistent AF patients after CPVA combined with linear ablation. SARA study demonstrated that CA was significantly more effective than antiarrhythmic drug therapy in maintaining sinus rhythm for patients with persistent AF, and it reduced the recurrence of sustained episodes (24 h) of AF by 47.4%.^6^ Furthermore, CA had a good safety profile according to the latest international registries.^15^ However, the energy from CA can aggravate the fibrosis of the LA muscle, and the scar after ablation can exacerbate the structural remodeling of left atrium and lead to the recurrence of AF. The efficiency of ablation is often offset by the recurrence of AF. Therefore, identifying non‐invasive indicators of LA remodeling to predict patients with high risk of recurrence after CPVA can help clinicians make more optimal treatment plans.^16^

Most studies on the risk factors of recurrence in persistent AF consider both persistent AF and long‐term persistent AF. Among trials that included patients with persistent AF or combined paroxysmal and persistent AF, the success rates after ablation ranged from 59 to 80% at 6 or 12 months.^17^, ^18^ The stratified studies on patients with persistent AF may be more accurate in predicting success rates. The patient selection in this study based on the concept of early persistent AF proposed by the 2017 HRS/EHRA/ECAS/APHRS/SOLAECE experts.^2^ Such patients are the main population of persistent AF patients that receive CA, and chronic AF patients benefit a lot from radiofrequency surgery.

The AF recurrence in patients with persistent AF after CA is largely determined by the changes in the structure and function of left atrium.^6^ The occurrence of AF changes the electrical and histological characteristics of the atrium. Electrical remodeling and structural remodeling of the atrium cause the vicious circle of “AF‐induced AF”.^19^ Under this condition, the frequency and duration of AF both increase, which maintains AF and transforms the disease to persistent AF. In patients with early persistent AF, the structural remodeling caused by degeneration and fibrosis of atrial myocytes can lead to the loss of active contraction function of left atrium and damaged storage function, which are manifested as decompensation of LA function. In recent years, many studies have shown that the degree of preoperative LA remodeling plays an important role in the success of CPVA.^16^ Many diagnostic techniques related to LA remodeling have been demonstrated to be helpful in predicting AF recurrence, including blood detection, echocardiography, computed tomography, and electrophysiological examination. The detection of blood and echocardiography are cost effective, convenient, and widely accepted in daily practice.^20^

It is feasible and practical to evaluate the risk of AF recurrence after ablation by analyzing the baseline level of relevant indicators. These indicators are easy to detect and can provide us with valuable information.^16^, ^17^, ^20^, ^21^ In patients with persistent AF and normal left ventricular function, BNP is mainly produced by LA myocytes. Meta‐analysis showed that the AF recurrence group had a higher pre‐ablation baseline level of BNP than sinus rhythm group, but the heterogeneity was also significant (I2 = 89%, *p* < .0001).^22^ In this study, the univariate ROC analysis showed that LA storage function (PI, DEI), LAAV, and plasma BNP could be used to predict the recurrence of early persistent AF after CPVA (AUC = 0.666–0.703), of which BNP had the best predictive performance (AUC = 0.703), with the sensitivity of 81.8%; however, the accuracy (61.9%) and specificity (55.0%)were not ideal. In addition, using the LA function parameters obtained by echocardiography to predict the recurrence of AF after radiofrequency has been explored by several groups in recent years. Left atrial function index (LAFI) is associated with AF recurrence after CA and has improved ability to predict AF recurrence as compared to the CHA_2_DS_2_‐Vasc score, especially among persistent AF patients.^23^ RT‐3DE is a new method of evaluating LA recurrence, and the automated measurements are comparable to CMR. This technique is highly reproducible and timesaving, and is highly sensitive to LA dysfunction in AF patients.^24^ Thus, the LAFI acquired by RT‐3DE may be a helpful tool for predicting AF recurrence. However, in this study, the AUC of DEI alone for predicting the recurrence of AF was only 0.666, which might be related to the fact that the LA function of patients with AF was easily affected by patient's heart function, heart rate, acquisition time and other factors.

The univariate association studies showed a predictive capacity but only moderately efficient. Thus, the risk assessment tools that combine with other prognostic indicators of AF recurrence may help refine the patient selection for radiofrequency catheter ablation.^25^ This study combined BNP level and LA function parameters to establish a new prediction model to predict postoperative recurrence. The model parameters included BNP level, LA storage function DEI and LAAV. RT‐3DE was used to obtain the preoperative time volume curve of the left atrium in patients with early persistent AF. The LA function parameters were obtained in the same cardiac cycle, and the LAAV obtained by TEE was used to exclude the impact of LA stunning on LA function during conversion of AF and evaluate LA function. Our study showed that patients with increased DEI, LAAV, and decreased BNP level were less likely to relapse, which is consistent with previous studies.^26^, ^27^ Therefore, this study included BNP level as an independent variable, combined with LA parameters to establish a new predictive model. ROC curve analysis confirmed that the multi‐factor combination model was better than single factor in the discrimination efficiency (AUC: 0.814 vs. 0.703, *p* = .026), and the improvement of diagnosis specificity and accuracy is more obvious than single factor.

This study still has some limitations. Due to the influence of heart rate during the examination and the various ventricular filling time, the data measured from patients with continuous AF may still be limited and biased, even though the data were measured and averaged from multiple cardiac cycles; Patients in this study were followed up for 3–6 months, and the recurrence of atrial fibrillation after 6 months was not included in this study. In addition, long‐term continuous record of patients' cardiac rhythm after ablation has not been carried out, and occasional atrial fibrillation may not be recorded, so it cannot reflect the long‐term specific results of patients with atrial fibrillation after ablation. Due to the small sample size, we used the Bootstrap internal verification method to verify the statistical effectiveness, and did not divide patients into modeling and verification groups or perform external verification. Therefore, it is necessary to further increase the sample size to optimize the prediction model parameters.

In summary, the prediction model of LA function combined with BNP level can be used to predict the recurrence of early persistent AF after CPVA, and the prediction efficiency, accuracy and specificity are better than a single factor model based on BNP. Therefore, the multi‐factor combination model can identify high‐risk patients for AF recurrence, help doctors optimize patient selection, inform patients about the risk–benefit ratio, guide operators to choose the best ablation strategy, and help apply personalized treatment plan.

## CONFLICT OF INTEREST

All authors declare that: (1) they have not received any support from any organization having an interest in the submitted works, whether financial or otherwise; (2) there are no other relationships or activities that may affect the submitted works.

## Data Availability

The data that support the findings of this study are available from the corresponding author upon reasonable request.

## References

[1] Chugh SS , Blackshear JL , Shen WK , Hammill SC , Gersh BJ . Epidemiology and natural history of atrial fibrillation: clinical implications. J Am Coll Cardiol. 2001;37:371‐378. 11216949 10.1016/s0735-1097(00)01107-4

[2] Calkins H , Hindricks G , Cappato R , et al. 2017 HRS/EHRA/ECAS/APHRS/SOLAECE expert consensus statement on catheter and surgical ablation of atrial fibrillation. Europace. 2018;20:e1‐e160. 10.1093/europace/eux274 PMC5834122 29016840

[3] January CT , Wann LS , Calkins H , et al. 2019 AHA/ACC/HRS focused update of the 2014 AHA/ACC/HRS guideline for the management of patients with atrial fibrillation: a report of the American College of Cardiology/American Heart Association task force on clinical practice guidelines and the Heart Rhythm Society. J Am Coll Cardiol. 2019;74:104‐132. 30703431 10.1016/j.jacc.2019.01.011

[4] Mark DB , Anstrom KJ , Sheng S , et al. Effect of catheter ablation vs medical therapy on quality of life among patients with atrial fibrillation: the CABANA randomized clinical trial. JAMA. 2019;13: 1275‐1285. 10.1001/jama.2019.0692 PMC6450275 30874716

[5] Gerhard H , Tatjana P , Nikolaos D , Elena A , Jeroen J , Carina Blomström‐Lundqvist , et al. 2020 ESC guidelines for the diagnosis and management of atrial fibrillation developed in collaboration with the European Association of Cardio‐Thoracic Surgery (EACTS). Eur Heart J. 2020;42: 373‐498. 10.1093/eurheartj/ehaa612 32860505

[6] Wynn GJ , Das M , Bonnettv LJ , Gupta D . Quality‐of‐life benefits of catheter ablation of persistent atrial fibrillation: a reanalysis of data from the SARA study. Europace. 2015;17:222‐224. 25028177 10.1093/europace/euu154

[7] Iwasaki YK , Nishida K , Kato T , Nattel S . Atrial fibrillation pathophysiology: implications for management. Circulation. 2011;124:2264‐2274. 22083148 10.1161/CIRCULATIONAHA.111.019893

[8] Mor‐Avi V , Yodwut C , Jenkins C , et al. Real‐time 3D echocardiographic quantification of left atrial volume: multicenter study for validation with CMR. JACC Cardiovasc Imaging. 2012;5:769‐777. 22897989 10.1016/j.jcmg.2012.05.011

[9] Rohner A , Brinkert M , Kawel N , et al. Functional assessment of the left atrium by real‐time three‐dimensional echocardiography using a novel dedicated analysis tool: initial validation studies in comparison with computed tomography. Eur J Echocardiogr. 2011;12:497‐505. 21685196 10.1093/ejechocard/jer066

[10] Zhang Y , Chen A , Song L , Li M , Chen Y , He B . Association between baseline natriuretic peptides and atrial fibrillation recurrence after catheter ablation. Int Heart J. 2016;57:183‐189. 26973277 10.1536/ihj.15-355

[11] Otani K , Takeuchi M , Kaku K , et al. Impact of diastolic dysfunction grade on left atrial mechanics assessed by two‐dimensional speckle tracking echocardiography. J Am Soc Echocardiogr. 2010;23:961‐967. 20667694 10.1016/j.echo.2010.06.023

[12] Verma A , Jiang CY , Betts TR , et al. Approaches to catheter ablation for persistent atrial fibrillation. N Engl J Med. 2015;372:1812‐1822. 25946280 10.1056/NEJMoa1408288

[13] Collins GS , Reitsma JB , Altman DG , Moons KG . Transparent reporting of a multivariable prediction model for individual prognosis or diagnosis (TRIPOD): the TRIPOD statement. BMJ (Clinical Research Ed). 2015;350:g7594. 10.1136/bmj.g7594 25569120

[14] DeLong ER , DeLong DM , Clarke‐Pearson DL . Comparing the areas under two or more correlated curves: a nonparametric approach. Biometrics. 1988;44:837‐845. 3203132

[15] Cappato R , Calkins H , Chen SA , et al. Updated worldwide survey on the methods, efficacy, and safety of catheter ablation for human atrial fibrillation. Circ Arrhythm Electrophysiol. 2010;3:32‐38. 19995881 10.1161/CIRCEP.109.859116

[16] Cameli M , Mandoli GE , Loiacono F , Sparla S , Iardino E , Mondillo S . Left atrial strain: a useful index in atrial fibrillation. Int J Cardiol. 2016;220:208‐213. 27389443 10.1016/j.ijcard.2016.06.197

[17] Berruezo A , Tamborero D , Mont L , et al. Pre‐procedural predictors of atrial fibrillation recurrence after circumferential pulmonary vein ablation. Eur Heart J. 2007;28:836‐841. 17395676 10.1093/eurheartj/ehm027

[18] Seow SC , Lim TW , Koay CH , Ross DL , Thomas SP . Efficacy and late recurrences with wide electrical pulmonary vein isolation for persistent and permanent atrial fibrillation. Europace. 2007;9:1129‐1133. 17923474 10.1093/europace/eum219

[19] Wijffels MC , Kirchhof CJ , Dorland R , Allessie MA . Atrial fibrillation begets atrial fibrillation. A study in awake chronically instrumented goats. Circulation. 1995;92:1954‐1968. 7671380 10.1161/01.cir.92.7.1954

[20] Jiang H , Wang W , Wang C , Xie X , Hou Y . Association of pre‐ablation level of potential blood markers with atrial fibrillation recurrence after catheter ablation: a meta‐analysis. Europace. 2017;19:392‐400. 27386883 10.1093/europace/euw088

[21] Bisbal F , Guiu E , Calvo N , et al. Left atrial sphericity: a new method to assess atrial remodeling. Impact on the outcome of atrial fibrillation ablation. J Cardiovasc Electrophysiol. 2013;24:752‐759. 23489827 10.1111/jce.12116

[22] Xu X , Tang Y . Relationship between brain natriuretic peptide and recurrence of atrial fibrillation after successful electrical Cardioversion: an updated meta‐analysis. Braz J Cardiovasc Surg. 2017;32:530‐535. 29267617 10.21470/1678-9741-2017-0008 PMC5731309

[23] Sardana M , Ogunsua AA , Spring M , et al. Association of Left Atrial Function Index with Late Atrial Fibrillation Recurrence after catheter ablation. J Cardiovasc Electrophysiol. 2016;27:1411‐1419. 27569695 10.1111/jce.13086 PMC5143220

[24] Tsang W , Salgo IS , Medvedofsky D , et al. Transthoracic 3D echocardiographic left heart chamber quantification using an automated adaptive analytics algorithm. JACC Cardiovasc Imaging. 2016;9:769‐782. 27318718 10.1016/j.jcmg.2015.12.020

[25] Njoku A , Kannabhiran M , Arora R , et al. Left atrial volume predicts atrial fibrillation recurrence after radiofrequency ablation: a meta‐analysis. Europace. 2018;20:33‐42. 28444307 10.1093/europace/eux013

[26] Inohara T , Kim S , Pieper K , et al. B‐type natriuretic peptide, disease progression and clinical outcomes in atrial fibrillation. Heart. 2019;105: 370‐377. 30228248 10.1136/heartjnl-2018-313642

[27] Xu M , Liu F , Ge ZX , Li JM , Xie X , Yang JH . Functional studies of left atrium and BNP in patients with paroxysmal atrial fibrillation and the prediction of recurrence after CPVA. Eur Rev Med Pharmacol Sci. 2020;24:4997‐5007. 32432763 10.26355/eurrev_202005_21191

